# Supplementary material for: Digital morphometry and cluster analysis identifies four types of melanocyte during uveal melanoma progression
Source: Commun Med (Lond). 2023 Apr 28;3:60. doi: 10.1038/s43856-023-00291-z (PMC10147908; doi:10.1038/s43856-023-00291-z)
Supplement: Supplementary file 1 — Supplementary Information [file 43856_2023_291_MOESM1_ESM.pdf]

Supplementary information to

Digital morphometry and cluster analysis identifies four types of melanocyte during uveal melanoma progression

Gustav Stålhammar[1,2], Viktor Torgny Gill[1,3]

[1]Department of Clinical Neuroscience, Division of Eye and Vision, Karolinska Institutet, Stockholm, Sweden.

[2]St. Erik Eye Hospital, Stockholm, Sweden.

[3]Department of Pathology, Vastmanland Hospital, Vasteras, Sweden.

Supplementary table 1. Cell segmentation settings

**Parameter**	**Setting**
Requested pixel size	0.5 μm
**Nucleus parameters**	
Background radius	8 μm
Median filter radius	0 μm
Sigma	2 μm
Minimum area	10 μm^2
Maximum area	200 μm^2
**Intensity parameters**	
Threshold	0.1
Max background intensity	2
**Cell parameters**	
Cell expansion	3.5 μm
Include cell nucleus	Yes
**General parameters**	
Smooth boundaries	Yes
Make measurements	Yes
**Intensity threshold parameters**	
Score compartment	Nucleus/cytoplasm as determined by type of stain
Threshold 1+	0.14
Threshold 2+	0.40
Threshold 3+	0.60
Single threshold	Yes

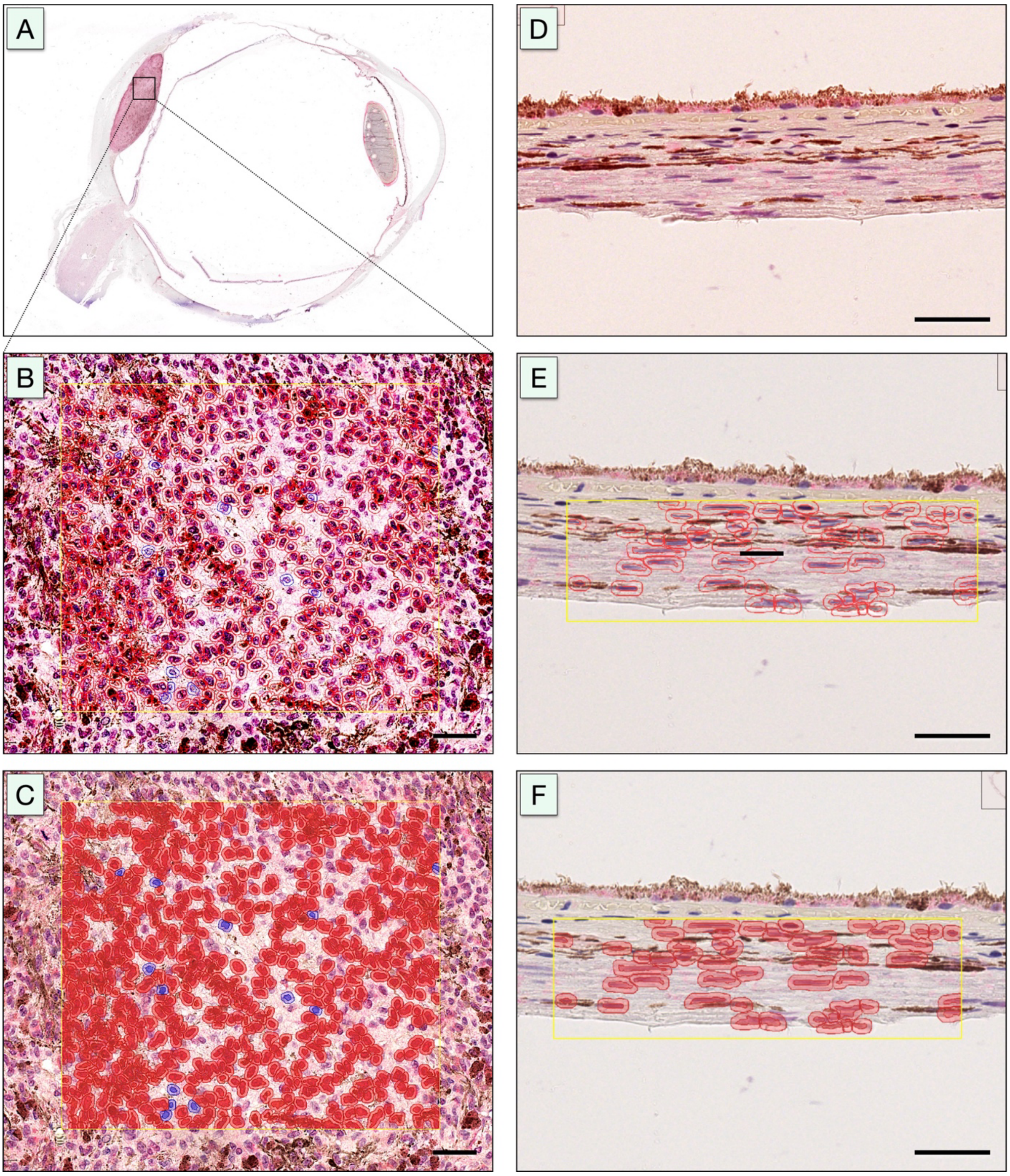

Supplementary figure 1. Example of cell segmentation. A) In this example, a BAP-1 stained choroidal melanoma is used to calibrate cell detection settings. B) In a randomly selected area of interest, the positive cell detection function has detected tumor cell nuclei. The cell expansion is adjusted until cytoplasms are captured appropriately. C) Positively stained cells (nBAP-1 positive) are marked red, and BAP-1 negative cells marked blue. D) A section of normal BAP-1 stained choroid. E and F) The same cell detection settings were found to delineate normal choroidal melanocytes appropriately. Scale bars: 40 µm

Supplementary table 2. Analyzed cell morphometric variables

Variable	Description and interpretation
Nucleus Area	Mean nucleus area, μm^2
Nucleus Perimeter	Mean nucleus perimeter, μm
Nucleus Circularity	Mean nucleus circularity. Compares the perimeter of a shape to the area it contains. The circularity of a circle is 1.00, and less for less circular objects.
Nucleus Max caliper	Mean nucleus length in longest dimension, μm
Nucleus Min caliper	Mean nucleus length in shortest dimension, μm
Nucleus Eccentricity	Mean nucleus eccentricity. A measure of how much the nucleus deviates from a spherical shape. A completely spherical nucleus has an eccentricity of 0.00, a nucleus with the shape of an elliptical 3D solid would have an eccentricity of 0.5, whereas a 3D conical distribution would have a value of 1.00.
Nucleus Hematoxylin OD mean	Mean nucleus hematoxylin staining intensity
nuclear BAP-1 (nBAP-1) mean OD	Mean staining intensity of BAP-1 in nuclei of melanocytes
Cytoplasm BAP-1 mean OD	Mean staining intensity of BAP-1 in cytoplasms
Cytoplasm IDO mean OD	Mean staining intensity of IDO in cytoplasms
Cytoplasm TIGIT mean OD	Mean staining intensity of BAP-1 in cytoplasms
Membrane IGF-1R mean OD	Mean staining intensity of IGF-1R in cytoplasms/cell membranes
Nucleus:Cell area ratio	Cell area divided by nucleus area

OD, optical density.

Supplementary table 3. Distribution of morphometric and staining characteristics across lesion types

	**Normal choroid *n*=7 cells=57 255**	**Choroidal nevi *n*=2 cells=18 276**	**Primary tumors *n*=17 cells=1 028 086**	**Metastases *n*=6 cells=141 794**	***P***
Area of the nucleus, mean μm³ (SD)	21.16 (13.40)	19.37 (10.45)	26.35 (14.23)	25.00 (13.96)	<0.0001†
Nucleus perimeter, mean μm (SD)	20.85 (7.66)	19.02 (6.54)	21.18 (6.55)	20.50 (6.42)	<0.0001†
Nucleus circularity, mean (SD)	0.63 (0.16)	0.69 (0.17)	0.73 (0.14)	0.74 (0.16)	<0.0001†
Nucleus max caliper, mean μm (SD)	8.43 (3.33)	7.50 (2.81)	7.98 (2.50)	7.68 (2.48)	<0.0001†
Nucleus min caliper, mean μm (SD)	3.67 (1.30)	3.70 (1.13)	4.62 (1.38)	4.55 (1.37)	<0.0001†
Nucleus eccentricity, mean (SD)	0.86 (0.13)	0.82 (0.14)	0.78 (0.14)	0.76 (0.15)	<0.0001†
Nucleus hematoxylin mean OD (SD)	0.27 (0.26)	0.34 (0.17)	0.47 (0.27)	0.39 (0.18)	<0.0001†
nBAP-1 mean OD (SD)	0.28 (0.33)	0.18 (0.14)	0.19 (0.23)	0.01 (0.10)	<0.0001†
Cytoplasm mean BAP-1 OD (SD)	0.37 (0.32)	0.18 (0.10)	0.33 (0.20)	0.12 (0.06)	<0.0001†
Nucleus to cell area ratio	0.17 (0.07)	0.19 (0.08)	0.28 (0.09)	0.23 (0.08)	<0.0001†
Membrane mean IGF-1R OD (SD)	-	-	0.20 (0.11)	-	-
Cytoplasm mean IDO OD (SD)	-	-	0.19 (0.17)	0.09 (0.13)	<0.0001‡
Cytoplasm mean TIGIT OD (SD)	-	-	0.32 (0.14)	0.24 (0.09)	<0.0001‡

OD, optical density. SD, standard deviation. †Kruskal-Wallis. ‡Mann–Whitney *U*.

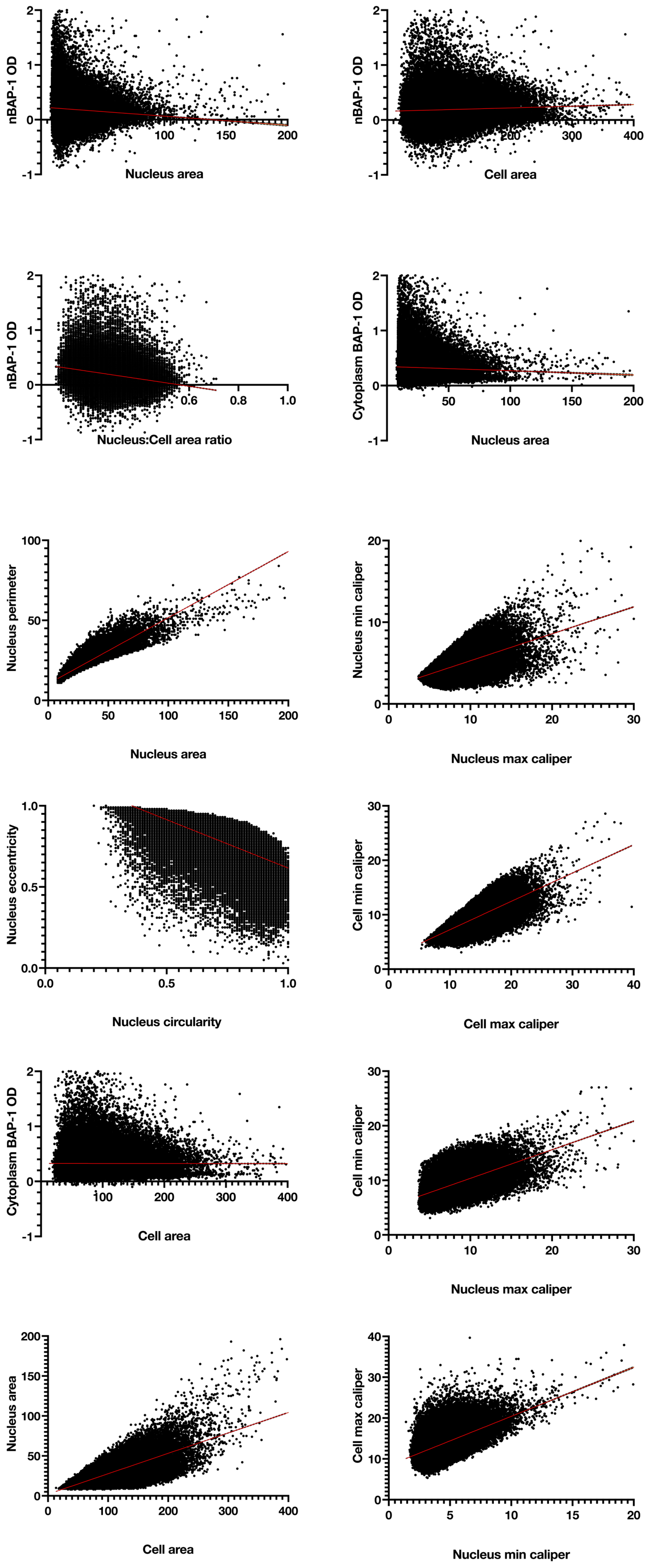

Supplementary figure 2. Scatter plots and linear regressions of morphometric and protein expression variables. OD, optical density.

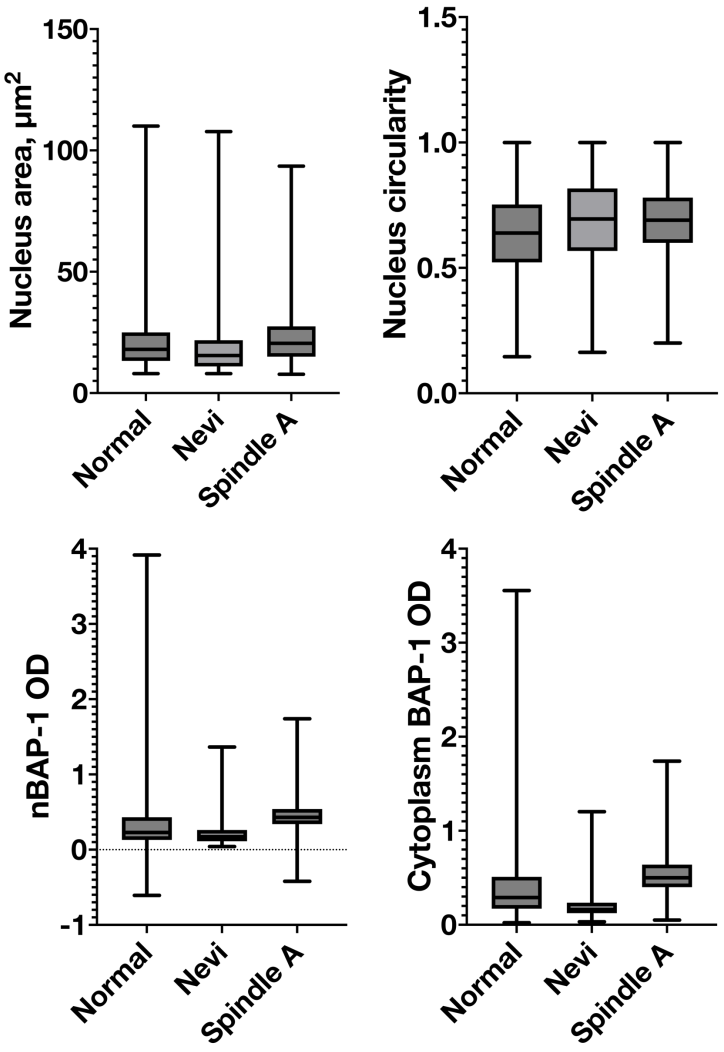

Supplementary figure 3. Area of the nucleus, nucleus circularity, nuclear BAP-1 optical density (nBAP-1 OD) and cytoplasmic BAP-1 OD across melanocytes in normal choroidal tissue (57 255 melanocytes from 7 enucleated eyes), in choroidal nevi (18 276 melanocytes from 2 enucleated eyes), and in uveal melanoma spindle A cells (236 460 tumor cells from 17 primary tumors). Spindle A cells had larger nucleus area, higher circularity, but lower expression of BAP-1 in nuclei and cytoplasms (Kruskal-Wallis $P<0.0001$). Centerlines denote median values, while the boxes contain the 25th to 75th percentiles of the datasets. Whiskers indicate minimum and maximum values.

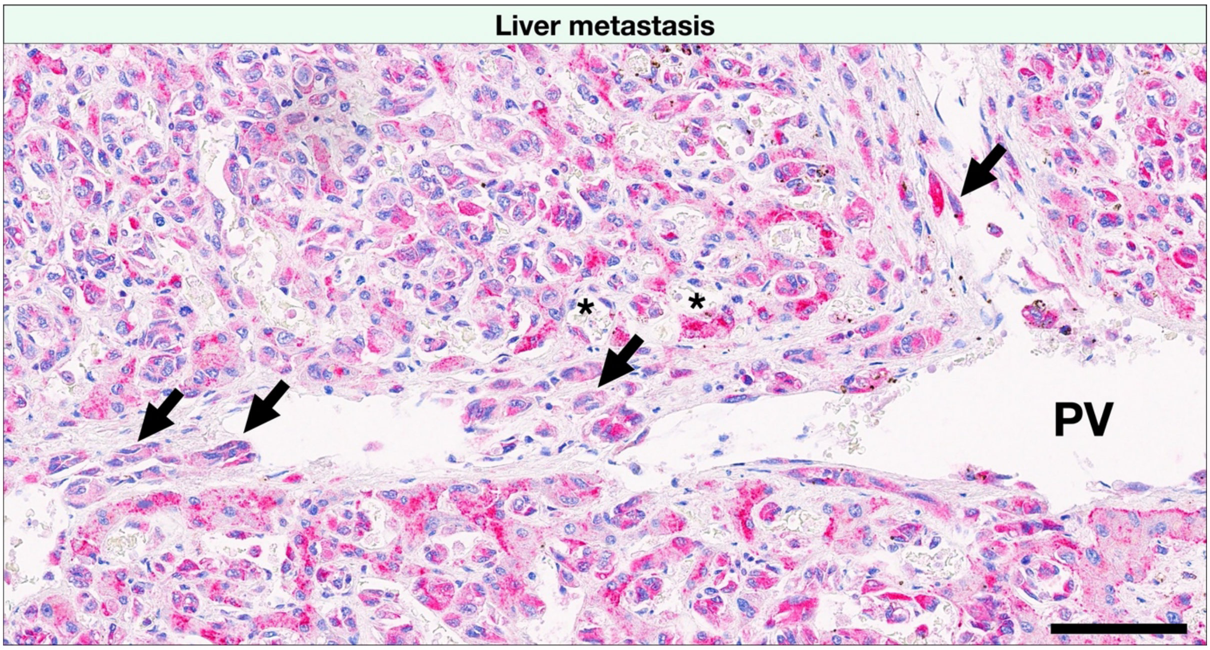

Supplementary figure 4. Liver metastasis growth pattern. Whereas large aggregates of extrasinusoidal metastatic cells most often represented the epithelioid cell type, metastatic tumor cells of the spindle B type (arrows) were more common in liver sinusoids and, as in this case, portal areas. BAP-1 staining product is accumulated in the cytoplasm of tumor cells, but no nuclear staining is seen. *bile ducts. PV, portal vein. Scale bar: 100 µm.
