# Supplementary material for: Digital morphometry and cluster analysis identifies four types of melanocyte during uveal melanoma progression
Source: Commun Med (Lond). 2023 Apr 28;3:60. doi: 10.1038/s43856-023-00291-z (PMC10147908; doi:10.1038/s43856-023-00291-z)
Supplement: Supplementary file 3 — Reporting Summary [file 43856_2023_291_MOESM3_ESM.pdf]

nature portfolio

Corresponding author(s): Gustav Stålhammar

Last updated by author(s): Mar 22, 2023

Reporting Summary

Nature Portfolio wishes to improve the reproducibility of the work that we publish. This form provides structure for consistency and transparency in reporting. For further information on Nature Portfolio policies, see our Editorial Policies and the Editorial Policy Checklist.

Statistics

For all statistical analyses, confirm that the following items are present in the figure legend, table legend, main text, or Methods section.

n/a	Confirmed	
☐	☒	The exact sample size (*n*) for each experimental group/condition, given as a discrete number and unit of measurement
☐	☒	A statement on whether measurements were taken from distinct samples or whether the same sample was measured repeatedly
☐	☒	The statistical test(s) used AND whether they are one- or two-sided<br>*Only common tests should be described solely by name; describe more complex techniques in the Methods section.*
☐	☒	A description of all covariates tested
☐	☒	A description of any assumptions or corrections, such as tests of normality and adjustment for multiple comparisons
☐	☒	A full description of the statistical parameters including central tendency (e.g. means) or other basic estimates (e.g. regression coefficient) AND variation (e.g. standard deviation) or associated estimates of uncertainty (e.g. confidence intervals)
☐	☒	For null hypothesis testing, the test statistic (e.g. *F*, *t*, *r*) with confidence intervals, effect sizes, degrees of freedom and *P* value noted<br>*Give P values as exact values whenever suitable.*
☒	☐	For Bayesian analysis, information on the choice of priors and Markov chain Monte Carlo settings
☐	☒	For hierarchical and complex designs, identification of the appropriate level for tests and full reporting of outcomes
☒	☐	Estimates of effect sizes (e.g. Cohen's *d*, Pearson's *r*), indicating how they were calculated

Our web collection on statistics for biologists contains articles on many of the points above.

Software and code

Policy information about availability of computer code

Data collection	No software was used
Data analysis	QuPath bioimage analysis<br>SPSS and Graphpad Prism statistical software

For manuscripts utilizing custom algorithms or software that are central to the research but not yet described in published literature, software must be made available to editors and reviewers. We strongly encourage code deposition in a community repository (e.g. GitHub). See the Nature Portfolio guidelines for submitting code & software for further information.

Data

Policy information about availability of data

All manuscripts must include a data availability statement. This statement should provide the following information, where applicable:

- Accession codes, unique identifiers, or web links for publicly available datasets
- A description of any restrictions on data availability
- For clinical datasets or third party data, please ensure that the statement adheres to our policy

The raw data generated in this study is published on figshare, 10.6084/m9.figshare.21360480

Human research participants

Policy information about studies involving human research participants and Sex and Gender in Research.

Reporting on sex and gender	Patient sex or gender was not considered in the design of this study. No clinical parameters including age, sex, gender, tumor size etc. is reported, beyond the measurement of tumor cells.
Population characteristics	Enucleated eyes from patients diagnosed with uveal melanoma were collected. No data on population characteristics were collected.
Recruitment	Enucleated eyes and liver metastases were identified in the archive of the St. Erik Ophthalmic Pathology Laboratory, Stockholm, Sweden. Inclusion criteria were: 1) Histologically proven uveal melanoma, 2) Paraffin block with enucleated eye or tissue from liver metastasis available, 3) Sufficient tissue for routine staining and immunohistochemistry. Exclusion criteria were: 1) Patient still alive, 2) Tissue older than 40 years, 3) Tumor tissue fragmented or affected by artefacts, 3) History of plaque brachytherapy, proton beam irradiation and/or transpupillary thermotherapy (TTT) prior to enucleation (for primary tumors), or 4) History of systemic chemotherapy, liver perfusion therapy (isolated hepatic perfusion, percutaneous hepatic perfusion or similar), immunotherapy, external beam irradiation or any other non-surgical treatment prior to tissue excision (for metastases). Metastatic tissue obtained at autopsy could be included on the same term that no non-surgical treatment for metastases had been given. The reason for exclusion criterion 3 and 4 is that such treatments may affect tumor histology. Normal choroidal melanocytes and choroidal nevi in the enucleated eyes were analyzed on the condition that they were separated from the tumor by at least 3 mm of histologically normal tissue, and were not affected by inflammation, edema, necrosis, bleeding, artefacts or similar.
Ethics oversight	The Swedish Ethical Review Authority

Note that full information on the approval of the study protocol must also be provided in the manuscript.

Field-specific reporting

Please select the one below that is the best fit for your research. If you are not sure, read the appropriate sections before making your selection.

☒ Life sciences ☐ Behavioural & social sciences ☐ Ecological, evolutionary & environmental sciences

For a reference copy of the document with all sections, see nature.com/documents/nr-reporting-summary-flat.pdf

Life sciences study design

All studies must disclose on these points even when the disclosure is negative.

Sample size	The sample size was determined by availability of archived tissue. A very large number of cells were analyzed (>1 million).
Data exclusions	No data was excluded
Replication	The measurements made are based on openly available software. We described how this software was used to allow for reproduciton by others.
Randomization	Not relevant. Cells were not grouped prior to analysis. Rather, we identified groups by objective cluster analysis, i.e. the software told us what the groups were and not vice versa.
Blinding	Blinding not relevant, as the groups were based on automatically generated clusters.

Reporting for specific materials, systems and methods

We require information from authors about some types of materials, experimental systems and methods used in many studies. Here, indicate whether each material, system or method listed is relevant to your study. If you are not sure if a list item applies to your research, read the appropriate section before selecting a response.

Materials & experimental systems

n/a	Involved in the study
☐	☒ Antibodies
☒	☐ Eukaryotic cell lines
☒	☐ Palaeontology and archaeology
☒	☐ Animals and other organisms
☒	☐ Clinical data
☒	☐ Dual use research of concern

Methods

n/a	Involved in the study
☒	☐ ChIP-seq
☒	☐ Flow cytometry
☒	☐ MRI-based neuroimaging

Antibodies

Antibodies used

Paraffin blocks were cut into 4 µm sections, pretreated in EDTA-buffer at pH 9.0 for 20 minutes and incubated with mouse monoclonal antibodies against BAP-1 (clone C-4, catalog no. sc.28383, Santa Cruz Biotechnology, Dallas, Texas, USA) and IDO (catalog no. 05-840, Sigma-Aldrich, Saint Louis, MO, USA), and rabbit monoclonal antibodies against IGF-1R (catalog no. ab39398, Abcam, Cambridge, UK) and TIGIT (catalog no. ab233404, Abcam). A red chromogen was used and sections were counterstained with haematoxylin and rinsed with deionized water. The deparaffinization, pretreatment, primary staining, secondary staining and counterstaining steps were run in a Bond III automated IHC/ISH stainer (Leica, Wetzlar, Germany). Dilutions between 1:20 and 1:1500 had been evaluated by a pathologist (GS), before selecting 1:40 for BAP-1, 1:1000 for IGF-1R and 1:75 for IDO and TIGIT.

Validation

All antibodies had been validated in humans before us, by the manufacturers as well as by independent research studies in humans.
